# Circadian Rhythm Disruption Was Observed in Hand, Foot, and Mouth Disease Patients

**DOI:** 10.1097/MD.0000000000000601

**Published:** 2015-03-13

**Authors:** Yu Zhu, Zhou Jiang, Guoguang Xiao, Suting Cheng, Yang Wen, Chaomin Wan

**Affiliations:** From the Department of Pediatrics, West China Second Hospital (YZ, GX, YW, CW); and Key Laboratory of Chronobiology, Ministry of Health, Sichuan University, Chengdu, PR China (ZJ, SC).

## Abstract

Hand, foot, and mouth disease (HFMD) with central nerve system complications may rapidly progress to fulminated cardiorespiratory failure, with higher mortality and worse prognosis. It has been reported that circadian rhythms of heart rate (HR) and respiratory rate are useful in predicting prognosis of severe cardiovascular and neurological diseases. The present study aims to investigate the characteristics of the circadian rhythms of HR, respiratory rate, and temperature in HFMD patients with neurological complications.

Hospitalized HFMD patients including 33 common cases (common group), 61 severe cases (severe group), and 9 critical cases (critical group) were contrasted retrospectively. Their HR, respiratory rate, and temperatures were measured every 4 hours during the first 48-hour in the hospital. Data were analyzed with the least-squares fit of a 24-hour cosine function by the single cosinor and population-mean cosinor method.

Results of population-mean cosinor analysis demonstrated that the circadian rhythm of HR, respiratory rate, and temperature was present in the common and severe group, but absent in the critical group. The midline-estimating statistic of rhythm (MESOR) (*P* = 0.016) and acrophase (*P* < 0.01) of temperature and respiratory rate were significantly different among 3 groups. But no statistical difference of amplitude in temperature and respiratory rate was observed among the 3 groups (*P* = 0.14). MESOR value of HR (*P* < 0.001) was significantly different in 3 groups. However, amplitude and acrophase revealed no statistical difference in circadian characteristics of HR among 3 groups. Compared with the common group, the MESOR of temperature and respiratory rate was significantly higher, and acrophase of temperature and respiratory rate was 2 hours ahead in the severe group, critical HFMD patients lost their population-circadian rhythm of temperature, HR, and respiratory rate. The high values of temperature and respiratory rate for the common group were concentrated between 3 and 9 pm, whereas those for the severe group were more dispersive. And the high values for the critical group were equally distributed in 24 hours of the day.

Circadian rhythm of patients’ temperature in the common group was the same as the normal rhythm of human body temperature. Circadian rhythm of patients’ temperature, HR and respiratory rate in 3 groups were significantly different.

This work was funded by grant 0040215501278 from the YiYao fund of Sichuan University.

## INTRODUCTION

Hand, foot, and mouth disease (HFMD) is a common disease induced by enterovirus for children, which is usually mild and self-limiting.^1^ However, complications including aseptic meningitis, brainstem encephalitis, acute flaccid paralysis, and pulmonary edema may occur in a small percentage of cases, and even pose a fatal risk.^2^

Symptom of lethargy was also identified as independent risk factor for neurological involvement (evident by cerebrospinal fluid pleocytosis), or severe HFMD.^3,4^ Clinical predictors for the risk of neurological involvement in 725 children with HFMD have been investigated. The results demonstrated that duration of fever ≥3 days, peak temperature ≥38.5°C, and history of lethargy were identified as independent risk factors for neurological involvement.^3,4^ We have also observed that HFMD patients with brainstem encephalitis had myoclonic jerks during sleep, which interfered with deep sleep, and caused children easily rouse from sleep. And these patients obviously looked lethargic and slept regardless of day and night during convalescent stage. Circadian rhythm describes biological phenomena that oscillate within a 24-hour cycle, which involves almost all aspects of human physiology, such as sleep–wake cycles and circadian rhythms of temperature.^5^ It has also been reported that circadian variations of heart rate (HR) and respiratory rate were correlated with cardiovascular diseases.^6,7^ The circadian rhythms of HR and respiratory rate are useful in predicting prognosis of severe cardiovascular and neurological diseases, such as acute myocardial infarction, ventricular arrhythmias and intracerebral hemorrhage.^8,9^ However, the circadian rhythms of HR and respiratory rate in HFMD patients have not been investigated. The aim of the present study was to determine the characteristics of the circadian rhythms of HR, respiratory rate, and temperature in HFMD patients.

## METHOD

### Participants

The participants enrolled in the study were from West China Second Hospital, Sichuan University in China between July 2008 and September 2011. HFMD patients were diagnosed according to the HFMD Diagnosis and Treatment Advices by Chinese Ministry of Health.^10^ The inclusion criteria were as follows: admitted patients with HFMD; patients were previously healthy children; and cases with complete medical records including individual information, clinical manifestation, and data of HR and respiratory rate for at least 24 hours. All HFMD cases meeting the inclusion criteria were enrolled in the study. Patients with underlying medical conditions, such as congenital heart disease, cerebral palsy, epilepsy, and other chronic nervous system diseases were excluded. Their basic characteristics and data of the clinical manifestations were measured and collected, such as symptoms of fever, rash, vomiting, myoclonic jerks, the time interval from onset to hospitalization, the time of fever, laboratory value for white blood cell count, and serum glucose.

### Study Protocol

This is a retrospective case analysis study that was approved by the Ethics Committee of West China Second Hospital, Sichuan University.

A total of 103 patients of HFMD were enrolled in the study. Their data were collected. They were assigned into 3 different groups according to their disease severity as follows: common group (n = 33), severe group (n = 61), and critical group (n = 9). A severe case was defined by the presence of more than one of the following complications of HFMD: encephalitis, aseptic meningitis, and acute flaccid paralysis,^1^ or at least 2 of the 4 clinical or laboratory investigations: duration of fever for 3 days or above, peak temperature ≥38.5°C; myoclonic jerks >3 times per hour, or other signs such as limbs weakness, limb jitter, or astasia; white blood cell count >17.5 cells/mm^3^; and blood glucose >8.3 mmol/L. Critical cases were those complicated with central cardiopulmonary failure, such as neurogenic pulmonary edema, pulmonary hemorrhage, neurogenic shock, or other critical conditions requiring endotracheal intubation and mechanical ventilation.^11^ And the rest of HFMD patients who were not categorized as severe or critical cases fell into the common group. The physicians in charge of the grouping program did not participate in the data collection and analysis.

### Measurement of HR, Respiratory Rate, and Temperature

HR and respiratory rate of the participants were measured by the multifunctional monitoring manufacture (Dash 3000, GE Healthcare, UT) during the first 48 hours in the hospital. Axillary temperature was measured by mercury thermometer for 3 to 5 minutes. HR, respiratory rate, and temperature were recorded every 4 hours.

### Analysis of Circadian Characteristics of HR, Respiratory Rate, and Temperature With Single Cosinor

The series of HR, respiratory rate, and temperature were analyzed with the least-squares fit of a 24-hour cosine function by the single cosinor method as previously described.^12,13^ If the characteristics of each series of HR, respiratory rate, and temperature fit the cosine curves (*P* < 0.05), it was defined as the presence of circadian rhythm. Otherwise, it was defined as the absence of the circadian rhythm. Results from each group were further analyzed by population-mean cosinor as previously described.^12–14^ Circadian rhythm characteristics were compared by parameter tests among the 3 groups.

### Statistical Analysis

Statistical analysis was performed on the SPSS System (version 19.0, SPSS Inc, Chicago, IL). Values were expressed as means ± SD or as percentages. Means were compared by the one-way analysis of variance test for independent samples. A *P* < 0.05 was considered statistically significant.

## RESULTS

### Characteristics of Participants

The ages of the overall patients ranged from 7 months to 6 years old (mean 2.33 years). The average age and gender ratios of the patients were no significantly different among the 3 groups (*P* = 0.408, *P* = 0.63 respectively). The time interval from onset to hospitalization was not statistically different in each group (*P* = 0.268), with an average interval of 3.33 ± 2.06 days.

Clinical characteristics of participants are shown in Table 1. The white blood cell count (*P* < 0.001) and serum glucose (*P* = 0.0002) value of the patients in the critical group were higher than those of severe and common groups. The predominant neurological presentations of the severe group were myoclonic jerks and tremors. Altogether, 15 patients were identified as aseptic meningitis (12 cases and 3 cases in the severe group and critical group, respectively), as shown in Table 2. Altogether, 7 patients in severe group (2/61) and critical group (5/9) suffered from acute flaccid paralysis. All patients in the critical group developed neurogenic shock, or pulmonary edema/hemorrhage. Two patients in the critical group died of pulmonary edema and cardiopulmonary failure, 41 h and 28 h after hospitalization, respectively. As shown in Table 2, there were 10 patients with neurologic dysfunction at discharge in severe group (3/61) and critical group (7/9), including 7 patients with limb weakness, 2 patients with eye movement disorders and 1 with bradycardia.

**TABLE 1:**
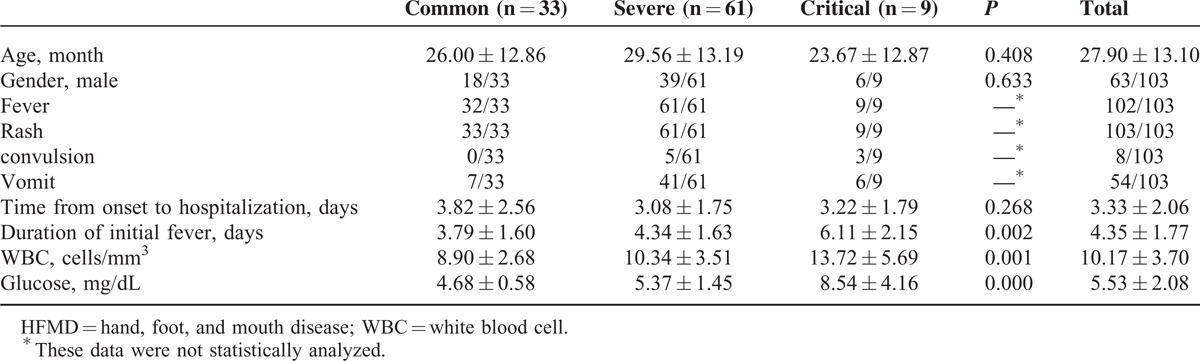
Clinical Characteristics of HFMD Patients

**TABLE 2:**
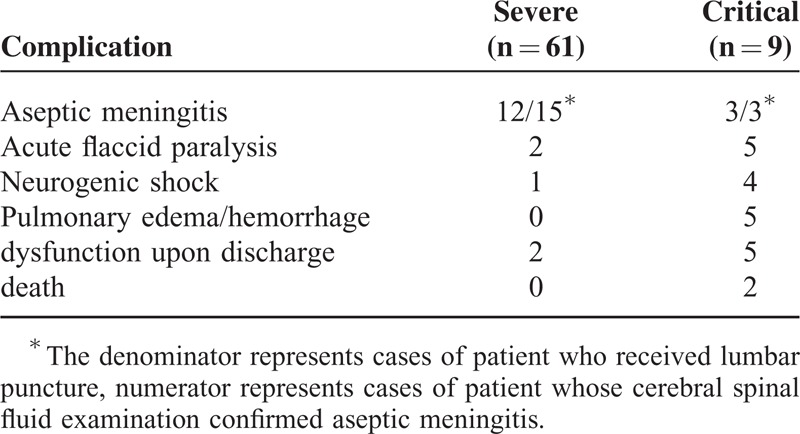
Complications in Patients of Severe or Critical Groups

### HR, Respiratory Rate, and Temperature

Single cosinor analysis showed that the circadian rhythms of temperature and respiratory rate were not detected with statistical significance in 11 of 33 patients of the common group. Circadian rhythms of HR were not detected with statistical significance in 17 of 33 patients of the common group. The circadian rhythms of temperature were not detected with statistical significance in 37 of 61 patients of the severe group. Circadian rhythms of HR were not detected with statistical significance in 29 of 61 patients in the severe group. And the circadian rhythms of respiratory rate were not detected with statistical significance in 41 of 61 patients in the severe group. The circadian rhythms of temperature were not detected with statistical significance in 7 of 9 patients in the critical group. Circadian rhythms of HR and respiratory rate were not detected with statistical significance in 3 of 9 patients in the critical group. Patients losing the circadian rhythm of temperature came to hospital significantly earlier than those who have rhythm in the severe and critical groups (*P* = 0.02).

**FIGURE 1:**
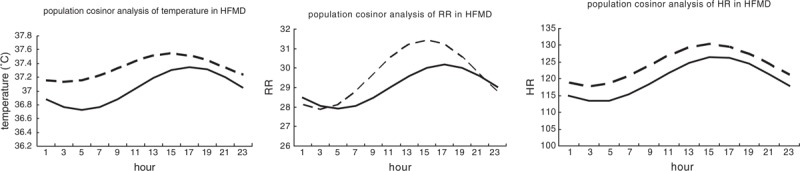
According to population-mean cosinor analysis, the cosine curves of solid line and dash line, respectively, represented cosine function equation of common group and severe group. Series data of the critical group cannot fit the cosine curve (*P* > 0.05). In the graph, midline-estimating statistic of rhythm (*P* = 0.016) and acrophase (*P* = 0.01) of temperature and respiratory rate had significant difference, but no statistical different amplitude of temperature and respiratory rate was observed (*P* = 0.14). Midline-estimating statistic of rhythm value of HR (*P* < 0.001) was significantly different, but amplitude and acrophase revealed no statistically significant difference. HFMD = hand, foot, and mouth disease, HR = heart rate.

**FIGURE 2:**
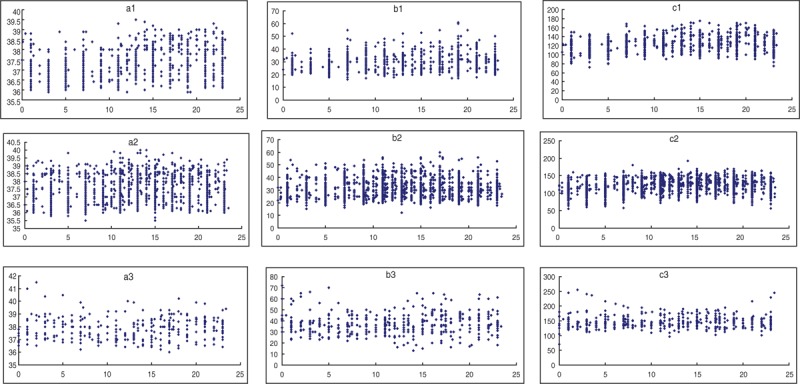
As shown A1, B1, And C1 represent series data of temperature, respiratory rate, and HR in the common group, respectively. A2, B2 And C2 represent series data of temperature, respiratory rate, and HR in the severe group, respectively. A3, B3 And C3 represent series data of temperature, respiratory rate, and HR in the critical group, respectively. Dots of scatter were actual data in each group. The high values of temperature and respiratory rate for the common group were concentrated between 3 to 9 pm, whereas those for the severe group were more dispersive. And the high values for the critical group were equally distributed in 24 hours of the day. Same as temperature, respiratory rate, and HR had differences in the 3 groups. HR = heart rate.

As shown in Table 3, population-mean cosinor analysis demonstrated that the circadian rhythm of HR, respiratory rate, and temperature was present in the common and severe group, but absent in the critical group. The midline-estimating statistic of rhythm (MESOR) (*P* = 0.016) and acrophase (*P* < 0.01) of temperature and respiratory rate had significant difference in 3 groups. But amplitude of temperature and respiratory rate had no statistical difference among the 3 groups (*P* = 0.14). Parameter tests of amplitude and acrophase revealed no statistical difference in circadian characteristics of HR among the 3 groups. According to population-mean cosinor, cosine curve of each group was shown in Figure 1. MESOR of the severe group was higher than that of the common group, with a 3 am nadir, a 3 pm zenith, and mean amplitude of 0.4°C. In temperature curve of the common group, value between peak and valley was 0.6°C, with a 5 am nadir, a 5 pm zenith. Temperature data of 3 groups were shown in Figure 2. And dots of scatter were actual data of temperature in each group. As shown in Figure 2, data of temperature above 38°C for the common group were concentrated in the time span between 10 am to 10 pm Most of the data for the severe group were concentrated in the same time span, although few between 1 to 5 am. Data of temperature above 38°C for the critical group, however, were equally distributed in 24 hours of the day. And what is more, a similar pattern was found in respiratory rate curve, temperature curve, and scatter graph.

**TABLE 3:**
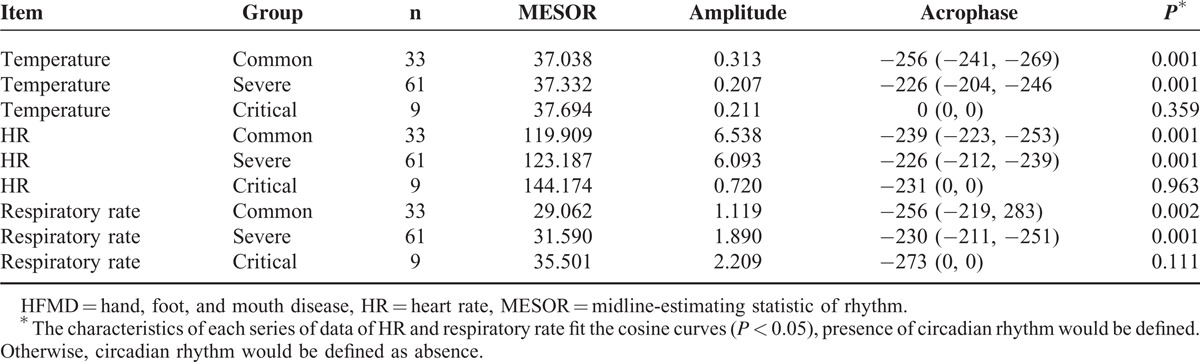
HR, Respiratory Rate and Temperatures of Patients With HFMD

## DISCUSSION

In the present study, the baseline characteristics of patients were consistent and comparable among the 3 groups. Moreover, age, time interval from onset to hospitalization, symptoms, and prognosis of these patients among the 3 groups were similar to previous studies.^1,11^

The circadian rhythms of HR, respiratory rate, and temperature were formed from the infantile period.^15^ The youngest patient in this study was 7 months old, and the age of the majority of cases was over 1-year old, in whom the circadian rhythm had already formed. In this study, it was a common phenomenon that circadian rhythms of temperature were lost in single cosinor analysis, especially in the patients of severe and critical groups. The time interval from onset to hospitalization of these patients losing rhythm of temperature was shorter than the patients having rhythm in the severe and critical groups. Many patients received antiviral therapy (either traditional Chinese medicine or western medicine), cortisone, or even immunoglobulin before admission. Thus, treatment before admission and duration of disease before admission may interfere with the rhythm analysis. And the same goes for rhythm analysis of HR and respiratory rate, too. Circadian rhythm disruption of temperature in single cosinor analysis of HFMD patients was very prominent, but not a sign to distinguish severity of disease.

Greg Kelly et al^16^ reviewed that normal rhythm of body temperature has a period of 24 hours, and nadir is between 3 and 6 am and the temperature apex is usually observed between 4 and 9 pm. Population-mean cosinor analysis in our study demonstrated that the characteristics for circadian rhythm of patients’ temperature in the common group were the same as those normal rhythms of human body temperature. Although the circadian rhythm of respiratory rate and temperature was present in the severe group, the MESOR and acrophase of temperature and respiratory rate were significantly different from the common group. The time at apex and nadir of temperature and respiratory rate in the severe group was 2 hours ahead. In the severe group, cosine curve of temperature looked flattened, whereas that of respiratory rate looked steep, but amplitude had no significant difference from that in the common group. From Figure 2, the high values of temperature and respiratory rate for the common group were concentrated between 3 to 9 pm, whereas those for the severe group were more dispersive. And the high values for the critical group were equally distributed in 24 hours of the day. Thijssen et al^17^ recently reported that a complete cervical spinal cord lesion showed the highest nocturnal mean core body temperature compared with able-bodied controls. It was believed that when the afferent and efferent arms of the sympathetic nerve system are lost, regulation of core body temperature was impaired.^17^ In fact, autonomic nervous system dysregulation, such as cold sweating, mottled skin, tachycardia, and hypertension was a critical stage before pulmonary edema in HFMD.^18^ There is a limitation of the study that data of temperature was not core body temperature, but axillary temperature. It needs further investigation to find out circadian rhythm disruption of core body temperature, for example, whether it could be used as an indicator for deterioration of HFMD or even involved in pathogenesis.

In conclusion, circadian rhythm of patients’ temperature in the common group was the same as the normal rhythm of human body temperature. Circadian rhythm of patients’ temperature, HR, and respiratory rate in 3 groups were significantly different.
